# Regional Alterations in Purkinje Cell Density in Patients with Autism

**DOI:** 10.1371/journal.pone.0081255

**Published:** 2014-02-24

**Authors:** Jerry Skefos, Christopher Cummings, Katelyn Enzer, Jarrod Holiday, Katrina Weed, Ezra Levy, Tarik Yuce, Thomas Kemper, Margaret Bauman

**Affiliations:** Department of Anatomy & Neurobiology, Boston University School of Medicine, Boston, Massachusetts, United States of America; Tokyo Medical and Dental University, Japan

## Abstract

Neuropathological studies, using a variety of techniques, have reported a decrease in Purkinje cell (PC) density in the cerebellum in autism. We have used a systematic sampling technique that significantly reduces experimenter bias and variance to estimate PC densities in the postmortem brains of eight clinically well-documented individuals with autism, and eight age- and gender-matched controls. Four cerebellar regions were analyzed: a sensorimotor area comprised of hemispheric lobules IV–VI, crus I & II of the posterior lobe, and lobule X of the flocculonodular lobe. Overall PC density was thus estimated using data from all three cerebellar lobes and was found to be lower in the cases with autism as compared to controls, an effect that was most prominent in crus I and II (p<0.05). Lobule X demonstrated a trend towards lower PC density in only the males with autism (p = 0.05). Brain weight, a correlate of tissue volume, was found to significantly contribute to the lower lobule X PC density observed in males with autism, but not to the finding of lower PC density in crus I & II. Therefore, lower crus I & II PC density in autism is more likely due to a lower number of PCs. The PC density in lobule X was found to correlate with the ADI-R measure of the patient's use of social eye contact (R^2^ = −0.75, p = 0.012). These findings support the hypothesis that abnormal PC density may contribute to selected clinical features of the autism phenotype.

## Introduction

Autism is a behaviorally defined neurodevelopmental disorder with core symptoms of impaired social interaction, delayed development of and qualitative abnormalities in communication, and restricted/repetitive and stereotyped behavior patterns [1]. Its wide range of additional associated symptoms and comorbidities has complicated efforts to determine the core neuropathological features of autism. Despite this clinical heterogeneity, numerous studies have described abnormalities involving the cerebellar circuitry and the limbic system [2]. Within the cerebellum, the consistently widely reported finding has been a decrease in the density of the Purkinje cells (PCs) [3]–[10], the large projection neurons in the cerebellar cortex. This is the first study, however, that was designed to precisely quantify regional alterations in PC density in autism and to test for association between PC density and a selection of relevant clinical behavioral measures.

**Figure 1:**
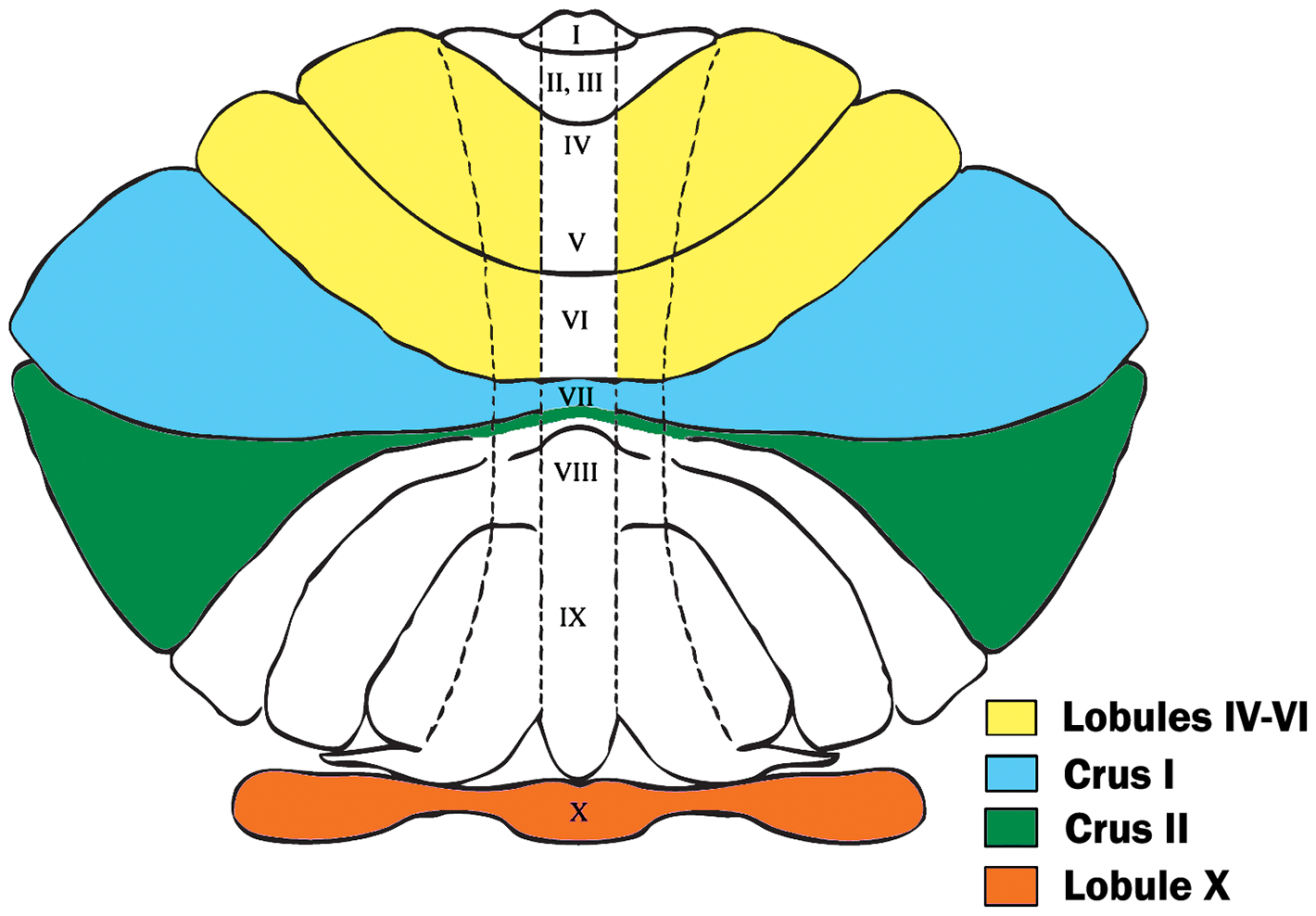
Cerebellar Regions Investigated. This flatmap diagram displays the four cerebellar regions of interest for our stereological assay. Hemispheric lobules IV–VI (in yellow) are bordered by the preculminate and superior posterior fissures, lateral to the fourth ventricle. Crus I (in blue) is the region bordered by the superior posterior and horizontal fissures. Crus II (in green) is bordered by the horizontal and ansoparamedian fissures. Lobule X, the flocculonodular lobe (in orange), is bordered by the posterolateral fissure. This image is an adaptation of the diagram by Larsell, 1958 [80].

In the present study, using a stereologic technique, we determined the PC densities in four cerebellar regions (Fig. 1). Two of these regions, crus I and II in the posterior lobe, were selected because previous studies have frequently noted abnormalities in these areas [3], [5]–[9]. Crus I and II are known to reciprocally connect with prefrontal cortical networks that modulate social behavior and behavioral planning [11], [12]. The third region, lobule X (the flocculonodular lobe), is associated with eye movement as well as vestibular regulation [13]–[18] and has been previously reported to display pathology in a subset of postmortem autistic cases [19], some of which were included in the present study [20], [21]. The fourth region, the hemispheric portion of lobules IV–VI, is a primary sensorimotor processing area that has been reported to undergo an age-dependent decline in PC density [22]–[24].

In an attempt to improve upon previous studies of PC density in autism, we sampled from a series of sections obtained throughout the entire cerebellum to measure PC density in regions within each of the three cerebellar lobes. In addition, the stereological methodology employed in this study significantly reduced the potential for variance in data acquisition due to subjective determinations, as compared with prior attempts to quantify PCs in autism [3]–[10], [25], [26]. With this methodology, we have attained a strong inter-rater reliability (>95% concurrence between the measurements of seven stereologists) and low variance in the data. Furthermore, the cases we have included in the autism group were selected to approximate the incidence of mental retardation (MR) and epilepsy in the overall autism population, which was not a feature of prior investigations and disallowed an assessment of the contribution of MR and epilepsy to the observation of lower PC density in autism in these studies [27], [28].

## Methods

### Case Demographics

The cerebella were obtained from the postmortem brains of eight individuals with autism and eight gender- and age-matched controls. Six of the cases were females (age range 4 to 21 years) and ten were males (age range 7 to 56 years), thus each group had 3 females and 5 males. All individuals in the autism group met DSM-IV and ADI-R criteria for autism spectrum disorders [1], [29]. The control cases had no known neurological disorder or known neuropathology. The clinical characteristics of both groups are summarized in Table 1. Postmortem interval (PMI), or time before initiating brain preservation, did not significantly differ between males and females nor between the autism and control groups. The majority of cerebella were sampled from the right hemisphere (88%), (Table 1). Three cases in the autism group were diagnosed with MR (IQ<70), ranging from mild to severe. Other symptoms in the autism group included: complications during pregnancy (38%), epilepsy (38%), delayed motor milestones (50%), delayed acquisition of verbal communication skills (88%), emotional disturbances such as depression and aggression (50%), and difficulty coordinating gaze (38%). Three cases in the autism group were reported to have suffered from developmental regression, two of whom had documented epilepsy. ADI-R scores did not significantly differ between males and females, but the male cases represented a wider age range (t = 2.35, df = 14, p = 0.036) than the female cases. The difference in average brain weight between males and females in our autism group was approximately twice the calculated difference for all autism cases cataloged in the Autism Tissue Program (ATP) database (327 vs. 186 grams) [http://www.atpportal.org], which may be a reflection of the small sample size available for this study. Based on the ATP data, cases diagnosed with autism display similar male-to-female differences in brain weight as controls, which is approximately a 10% increased fresh brain weight in males [30]. Due to this divergence from the normative data (in which we noticed an approximate 20% difference in our samples between the male and female autism cases), we have tested our analyses of PC density for covariance with brain weight and cerebellar volume (as described below in Statistical Analyses).

**Table 1:** Clinical Characteristics.

Case	Diagnosis	Sex	Age	Brain Weight^a^	PMI^b^	MR^c^	Epilepsy	Regression	Cause of Death
B-6115^d^	Autism	F	17	1158	25	no	no	no	Dilated cardiomyopathy
B-6403	Autism	M	7	1610	25	no	yes	yes	Drowning
UMB-1627	Autism	F	5	1390	13.3	no	no	no	Auto trauma
IBR-93-01	Autism	M	23	1610	14	no	yes	no	Drowning
B-6276	Autism	M	56	1570	3.4	moderate MR	no	no	Arteriosclerotic heart disease
B-6212	Autism	M	36	1480	24	severe MR	no	yes	Circulatory failure, renal failure
UMB-1638	Autism	F	21	1108	50	mild MR	yes	yes	Obstructive pulmonary disease
B-5666	Autism	M	8	1570	22.2	no	no	no	Sarcoma
UMB-1843	Control	F	15	1250	9	no	no	N/A	Multiple Injuries
UMB-1846	Control	F	20	1340	9	no	no	N/A	Multiple Injuries
UMB-4898	Control	M	7	1240	12	no	no	N/A	Drowning
B-6736	Control	F	4	1530	17.0	no	no	N/A	Acute broncho-pneumonia
UMB-1646	Control	M	23	1520	6	no	no	N/A	Ruptured spleen
BTB-3983	Control	M	52	1430	12.5	no	no	N/A	Atherosclerotic cardiovascular disease
UMB-1576	Control	M	32	-	24	no	no	N/A	Compressional asphyxia
IBR-252-02^d^	Control	M	51	1450	18	no	no	N/A	Myocardial infarct

a Fresh brain weight (in grams) was measured prior to tissue processing.

b Postmortem interval.

c Mental Retardation.

d Left brain hemisphere was investigated. (All other cases were investigated from the right brain hemisphere).

### Tissue Preparation

This study utilized prepared cerebellar histological sections obtained from the ATP. These sections are a component of the ATP's Brain Atlas Project, which is a multi-site collaborative investigation of an established cohort of identically processed cases that agreed to donate their brain tissue for autism research (http://www.autismtissueprogram.org).

**Figure 2:**
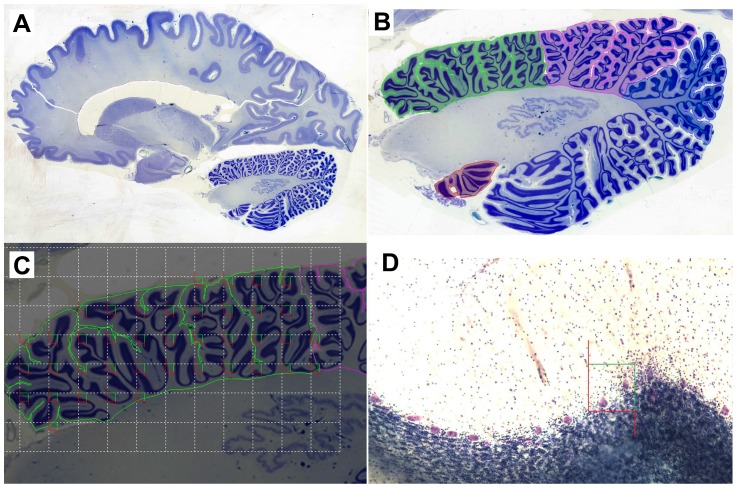
Stereological Assessment of PC Density. This figure depicts our stereological approach for quantifying PC density in each region of the cerebellum. 2a displays a full sagittal section of the human brain. 2b represents the positioning of contour lines used to select the boundaries around each region of interest, within which sampling occurs. Lobules IV–VI are highlighted in green, crus I is highlighted in pink, crus II is highlighted in blue, and the flocculus of lobule X is highlighted in orange at the bottom of the section. 2c displays a hypothetical placement of counting frames (optical disector probes) within lobules IV–VI. The counting frame size has been increased to aid in visualization for this example. The white dotted line displays the randomly imposed grid over the contour, which regulates the distance between counting frames along the X–Y plane of the section. A counting frame is placed in the top-left of each grid cell if it will include a portion of the region of interest as designated by the contour line. Finally, 2d displays a histological section with an imposed counting frame that includes two PCs to be counted. The use of the optical disector probe dictates that PCs are counted when within the counting frame or at all touching the green line, but never when cells intersect with the red line.

Tissue prepared for the Brain Atlas Project was processed by the New York State Institute for Basic Research in Developmental Disabilities (NYS-IBR) following protocols approved by their Institutional Review Board [21]. Whole fresh brain weights were obtained prior to processing. Each brain was cut mid-sagittally through the corpus callosum and brainstem. One brain hemisphere was fixed in 10% buffered formalin, and the other brain hemisphere was frozen. Following at least 3 weeks of fixation, magnetic resonance imaging (MRI) scans were acquired of the fixed brain hemispheres from the cases in the autism group using a 1.5 T GE Signa Imager (General Electric, Milwaukee, USA). T1-weighted, fast gradient echo MRI was used to sample each brain in 1.5 mm-thick virtual slices in the coronal plane, perpendicular to the anteroposterior axis of the hippocampus (FOV = 25 cm, NEX = 1, matrix = 256×192, TR = 35 ms, FA = 60°). Following imaging, each fixed hemisphere was washed overnight in water and subsequently dehydrated in increasing ethanol concentrations (50% for 3 days, 70% for 4 days, 80% for 3 days, and 95% for 4 days). The tissue was then embedded in 8% celloidin [31]. Celloidin blocks were hardened in chloroform vapor for approximately 2.5 weeks, and then stored in 70% ethanol. A series of serial 200 µm sections separated by 1.2 mm increments (every 6^th^ section) was obtained throughout the entire brain hemisphere as well as through the brainstem and cerebellum of each case (Fig. 2a). The sections were immersed in water for 2–3 hours, after which they were Nissl-stained with cresyl violet and mounted with Acrytol. Each case was assigned a brain bank identification number to maintain donor anonymity.

### Stereological Methodology

Using two cerebellar atlases [32], [33], the cerebellar regions were anatomically defined as follows: crus I is bordered by the superior posterior and horizontal fissures; crus II is bordered by the horizontal and ansoparamedian fissures; lobule X includes the nodulus and flocculus which together are bordered by the posterolateral fissure; and hemispheric lobules IV–VI are bordered by the preculminate and superior posterior fissures, lateral to the fourth ventricle (Fig. 1).

To facilitate unbiased quantification of PCs within each selected cerebellar region, the optical disector probe [34], a 3D counting frame, was employed throughout the entire series of cerebellar slides (approximately 40 slides per case), such that we sampled throughout the entirety of each region of interest (Fig. 2). Each cerebellar lobule was sampled throughout the cortex and adjacent white matter (Fig. 2b). Slides were analyzed using a Nikon Eclipse 80i microscope equipped with a motorized stage (Ludl Electronic Products, Hawthorne, NY) and microcator (a positional deviation meter, Heidenhain, Schaumburg, IL) for precise navigation in the XY and Z planes of the section, respectively. The microscope was guided by a computer with the Stereoinvestigator 10 (MBF Bioscience, Williston, VT) software package calibrated to move the slide with 1 µm precision. This system ensured systematic, uniform and random sampling. Preliminary measures were taken to ensure >95% inter-rater reliability (number of stereologists = 7) and <0.07 coefficient of error (CE) for each region investigated (Gundersen CE, m = 1. For a detailed description: [35]). Our counting object was the PC soma, which was readily identifiable with a 40× objective lens (NA = 0.75), and thus PCs were counted when the borders of the soma were in focus. Slides were viewed through the microscope eyepiece as well as on the computer monitor using an Optronics Microfire digital camera. The ATP Brain Atlas celloidin series is comprised of 200 µm sections designed to aid in volumetric analyses. However, PCs were most reliably identifiable through a depth of the first 80 µm in each section due to excessive light diffraction at greater depths. Therefore, we employed a 5 µm guard volume (to prevent counting error due to cell loss during tissue sectioning) and sampled through 75 µm below the guard volume. The computer software ensured that the 19,600 µm^2^ counting frames were randomly imposed and evenly spaced within the region of interest on each slide (Fig. 2c). The distance between the counting frames was determined separately for each cerebellar region to ensure that optimal sampling parameters were employed. Sampling parameters were considered optimal if they minimized systematic error from oversampling, while still providing reliably reproducible estimates of PC density, as determined by the CE [36]. Using optimized parameters, a counting frame was placed every 2500 µm^2^, 2200 µm^2^, 400 µm^2^, and 1400 µm^2^ along the X and Y axes, in crus I, crus II, lobule X, and hemispheric lobules IV–VI, respectively. As a result, the mean number of counting frames for each of these four regions was: 1106, 818, 1392, and 1690, respectively. Overall, approximately 5,000 counting sites throughout the three cytoarchitectonic layers of the cortex and the associated underlying white matter were assayed in each cerebellum (Fig. 2). Using these parameters, approximately 450 PCs were counted in each cerebellar region with an average CE of 0.053.

In some cases, less than 100% of each region of interest was available, which precluded the possibility of obtaining estimates of total PC number. Case notes indicated that as much as 10% of the tissue had been lost during processing. In addition, some slices demonstrated fraying at the edge of the folia. In a few instances, these complications prevented us from obtaining estimates from each target region and thus only the regions that could be completely sampled were analyzed. As a result, rather than estimating total PC number, data were analyzed as PC density measurements, calculated by dividing the total number of PCs counted in a region by the summed volume of each counting box placed in that region (Eq. 1).
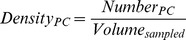
(1)


### Additional Volumetry Using MRI

We were able to collect a measure of total cerebellar volume for each case in the autism group by performing planimetry on the MRI data obtained by the NYS-IBR using the formalin fixed brains. Tissue shrinkage during fixation ranged from 44% to 52% and did not differ significantly between the autism and control groups. Planimetry was performed with ImageJ [http://rsbweb.nih.gov/ij/] using the Yawi3D plugin [http://yawi3d.sourceforge.net], which generates an automated selection of the region of interest on each MRI slice. Following manual adjustment using a pen tablet interface (Wacom, Otone, Japan) to ensure precise delineation of cerebellar boundaries, the cerebellar area in each slice was calculated and summed. As the MRI acquisition and tissue processing protocols used in this study were standardized across samples, it was possible to make comparisons within the autism group using the planimetric data for each case. However, since control brains in the Brain Atlas Project lacked MRI data, group comparisons were not made. Obtaining total cerebellar volume measurements for the cases in the autism group allowed for an assessment of the contribution of overall tissue volume to PC density in each region, particularly as it pertained to gender differences within the autism group, as well as the correlation between PC density and behavioral measures.

### Statistical Analyses

To assess the differences between group means on the basis of gender and autism diagnosis, we performed a linear mixed model test fitted by maximum likelihood, with weighted least squares (WLS) correction based on the regional volume from which each PC density measure was obtained. To test for regional differences in PC density as an effect of gender and autism diagnosis, we repeated the test without the WLS correction. The above tests were also repeated with brain weight as a covariate to investigate the contribution of brain weight to our findings (Table 2). False discovery rate controlled t-tests [37] were then performed to inspect the interaction between gender, diagnosis, and region. The appropriate t-test was chosen based on the results of Levene's test of equality of variances between each group. PC density measures in each region were tested for correlation with the non-parametric scores in each domain of the ADI-R (communication, social interaction, and restricted/repetitive behavior), as well as with the 7 specific questions in the ADI-R recently identified by machine learning analyses to be most indicative of an autism diagnosis [Presentation by Dennis Wall, “Shortening the Behavioral Diagnosis of Autism Through Artificial Intelligence and Mobile Health Technologies,” Autism Consortium 2011 Symposium, Boston, MA]. (For a detailed description of the statistical methods used to select these specific behavioral variables, Wall et al. has recently published a similar analysis of the Autism Diagnostic Observation Schedule [38]). For all tests of correlation involving non-parametric behavioral measures, Spearman rank correlations were performed. To assess the probability of a false positive correlation, the false discovery rate procedure was performed. The effects of potentially confounding factors or clinically relevant covariables were investigated. Parametric variables such as age, brain weight, cerebellar volume, PMI, and fixation time were tested for covariance with PC density estimates using univariate generalized linear models. To test the effects of clinically relevant factors reported in the case histories of the autism group, such as epilepsy, diagnosis of MR, developmental regression, lack of verbal development, delay in attaining motor milestones, and complications during pregnancy, separate t-tests were performed for each cerebellar region. Additionally, for cases in the autism group, linear regression analysis was performed to detect potential associations between PC density and the age at which the subject first walked unaided.

**Table 2:** Linear Mixed Models.^a^

		1		(2)		3		(4)	
		F	p-value	F	p-value	F	p-value	F	p-value
**Variables**	diagnosis	**6.050**	**0.020**	*7.515*	*0.010*	*7.407*	*0.015*	*9.583*	*0.007*
	gender	**4.802**	**0.036**	**3.697**	**0.068**	*6.373*	*0.022*	*4.423*	*0.054*
	region	*7.159*	*0.001*	*6.652*	*0.001*	**33.783**	**0.000**	**36.031**	**0.000**
**Interactions**	diagnosis * gender	**0.014**	**0.905**	**0.214**	**0.648**	*0.000*	*1.000*	*0.340*	*0.568*
	diagnosis * region	*0.965*	*0.418*	*1.414*	*0.253*	**0.380**	**0.768**	**0.401**	**0.753**
	gender * region	*2.281*	*0.093*	*3.171*	*0.035*	**2.093**	**0.115**	**2.291**	**0.093**
	gender * diagnosis * region	*1.615*	*0.200*	*2.013*	*0.128*	**3.946**	**0.014**	**5.078**	**0.005**
**Covariables**	brain weight^a^	N/A		0.372	0.551	N/A		0.311	0.585
**Residual Weight**		Regional volume		N/A		Regional volume		N/A	
**Number of Cases**		16		15		16		15	
**Diagnosis & Gender**	**(M/F)**	Autism (5/3)Control (5/3)		Autism (5/3)Control (4/3)^b^		Autism (5/3)Control (5/3)		Autism (5/3)Control (4/3)^b^	

a Cells in the table that have bold data presented designate the target results for each variable and interaction, whereas cells with italicized data designate results that are more adequately assessed by a different test presented in the table: Test 1 was designed to investigate the overall effect of gender and autism diagnosis on PC density. It incorporated cerebellar regional volume as a WLS weight to adjust the test's significance relative to the regional volume for each PC density measurement. Test 3 was designed to test the regional differences in PC density as an effect of gender and diagnosis. Tests 2 and 4 are designed to investigate the contribution of fresh brain weight (grams), as a correlate of tissue volume [39], to Tests 1 and 3, respectively.

b One control male case was missing brain weight information.

Logarithmic transformation was performed on all PC density estimates to adjust for skew from a normal distribution. The significance of each finding was only marginally affected by this transformation. Because our PC measures were obtained from each lobe of the cerebellum (anterior, posterior, and flocculonodular), we generated an estimate of overall cerebellar PC density as a composite of the regional volume-weighted mean PC densities. Therefore, PC density estimates from larger cerebellar regions (crus I & II) had a greater impact on this overall PC density estimate (similar to the WLS correction in the linear mixed models described above). Cerebellar regional volumes used in this overall PC density estimate were based on planimetric measurements obtained from the histological sections during stereological PC density estimation.

All statistical tests were two-sided, with an alpha level of 0.05, and false discovery rate was used to adjust the cutoff for significance as mentioned in the Results. Confidence intervals were set at 95% for all comparisons, and for correlations, these intervals were determined using bootstrapping with 1,000 replications. Statistical computations were performed with SPSS Version 19 (IBM, Armonk, NY).

## Results

### Comparisons Based on Diagnosis

#### Autism cases demonstrate a lower overall Purkinje cell density

We pooled all PC density measurements for each case and tested for an overall difference in PC density based on autism diagnosis using a linear mixed model. The model clustered the PC density measures from each case, and weighted the significance of each region's PC density measures in proportion to the respective region's volume (thereby avoiding the overrepresentation of smaller regions in the overall analysis). The regional volume estimates utilized in this analysis were calculated from histological area measurements of each region obtained as the PCs were quantified on each slide.

Utilizing this model, we observed an effect of autism diagnosis on overall PC density (p = 0.02, Table 2, Test 1). This was reflected as an 11% lower regional volume-weighted mean PC density in the autism group. This finding was additionally corrected for covariance with each case's brain weight, which only slightly improved the significance (p = 0.01, Table 2, Test 2). Because brain weight is a correlate of brain volume [39], this finding indicates that the difference in PC density between the autism and control groups may not be due to volumetric differences but may rather reflect a difference in overall PC number. An alternative possibility is that regional volumetric changes occur in autism (that may not make a substantial impact on overall brain weight but could still affect regional PC density), but a linear mixed model designed to test this found no effect of diagnosis on regional volume (p = 0.936).

#### Purkinje cell density is more affected in crus I & II in autism

**Figure 3:**
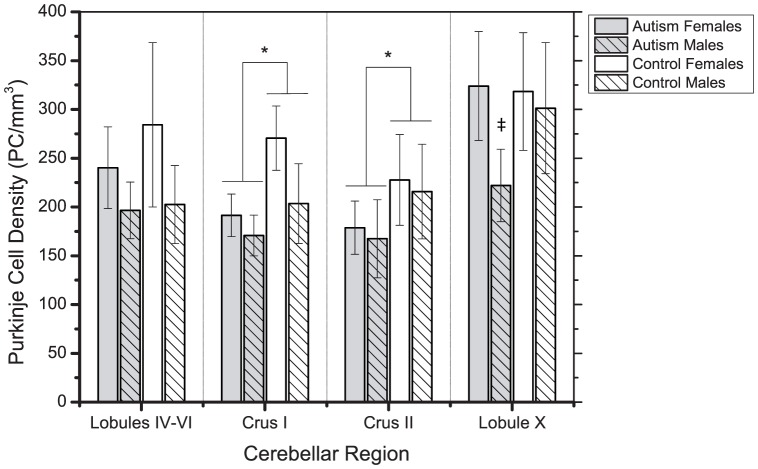
Regional PC Density. This graph demonstrates the findings from our linear mixed models (Table 2). Mean PC density was lower in the autism group in each region assayed, but this finding was most prominent in crus I and II (marked with *), where there was 19.8±9% and 21.7±9% lower PC densities, respectively (p = 0.039 and p = 0.032, t-test). (See the results of this test in Table 3). Lobule X PC density was lower in the males with autism (marked with ‡) than in control males (p = 0.05), and lower than female cases in the autism group (p = 0.022). Bars represent mean ± standard deviation. The number of subjects for each test from each diagnostic group and their gender are represented in the results tables (Tables 2 and 3).

Linear mixed models were used to test for an interaction between autism diagnosis and regional PC density (Table 2, Tests 3 & 4). These tests indicated that autism did not affect any of the four regions' PC densities differentially (p = 0.768, Table 2, Test 3). Indeed, each region demonstrated a lower PC density in the autism cases compared to controls. Furthermore, we found that the densities of some of the regions were highly correlated: crus I & II exhibited the strongest correlation (R^2^ = 0.832, p = 1.2×10^−4^). When the effect of gender was incorporated, however, we did observe a three-way interaction between gender, diagnosis, and region (p = 0.014, Table 2, Test 3). This indicates that autism affects each region differently in males compared to females. Figure 3 demonstrates these regional effects, and shows that in crus I & II, both males and females displayed a lower PC density in the autism group.

Our *a priori* hypothesis based on previous studies was that PC density would be most affected in crus I & II in autism [3], [5]–[9]. To investigate this, we proceeded with *post hoc* analyses of each region's PC density with respect to autism diagnosis using t-tests controlled for false discovery rate. These tests demonstrated that the major effect of autism diagnosis on PC density was in crus I & II, which demonstrated an approximately 20% lower PC density (p = 0.039 & p = 0.032, Fig. 3, Table 3).

**Table 3:** Regional Differences in Purkinje Cell Density.

Region	t	df	p-value	Austism (M/F)	Control (M/F)
Lobules IV–VI	0.677	11	0.512	6 (4/2)	7 (4/3)
Crus I	2.293	13	0.039^a^	8 (5/3)	7 (5/2)
Crus II	2.407	13	0.032^a^	8 (5/3)	7 (5/2)
Lobule X	1.44	13	0.173	8 (5/3)	7 (5/2)

a Met the cutoff for false discovery rate.

#### Age-related decline in Purkinje cell density does not differ in autism

**Figure 4:**
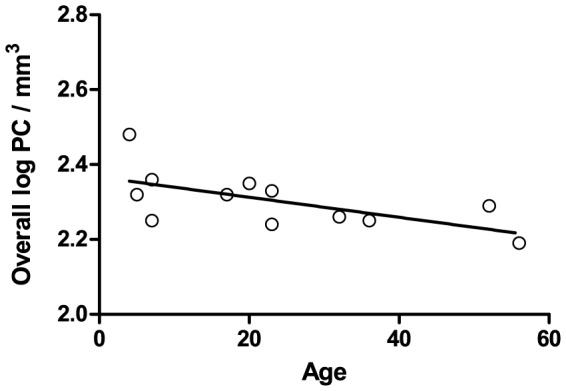
Overall PC Density Decreases with Age. Overall PC density obtained from the anterior, posterior, and flocculonodular lobes negatively correlates with age (R^2^ = −0.39±0.14, p = 0.030). Some cases were missing data from an individual region (as described in Methods) and were not included: one female and one male case from the autism and control groups were not included.

Prior investigations have reported evidence of an age-related decline in PC density in the anterior lobe [22]. Our PC density measures from the hemispheric portions of lobules IV–VI displayed a trend in support of these previous observations that did not reach significance (R^2^ = −0.23±0.16, p = 0.097). However, the age range of our samples did not extend as far into old age as in the prior reports. If samples from individuals with more advanced age were included in the present investigation, it is possible we would have observed a more significant correlation between age and PC density in this region. Nonetheless, we did observe an age-related decline in overall PC density (R^2^ = −0.39±0.14, p = 0.030) (Fig. 4) that did not differ between the autism and control groups.

#### Flocculonodular dysplasia does not impact PC density

All of the cases in our autism group have been analyzed by neuropathologists at the NYS-IBR in a manner that was blind to diagnosis. Five cases in our study (63%) were reported to display flocculonodular dysplasia by Wegiel et al. [21]. However, we found that lobule X dysmorphology had no effect on the PC density in this region (p = 0.662).

### Comparisons Based on Gender

#### Males have a lower overall Purkinje cell density than females

We investigated the effect of gender on overall PC density by using the same linear mixed model discussed above (Table 2, Test 1 & 2). Again, this test incorporated all PC density measures, clustered by case, and the significance of each measure was weighted by the volume of the region from which it was taken. Using this approach, we observed an effect of gender on overall PC density (p = 0.036, Table 2, Test 1). This was reflected as a 21% lower regional volume-weighted mean PC density in the male cases compared to the females.

It was particularly important to correct this model for covariance with brain weight (Table 2, Test 2), because our female cases demonstrated a 13.5% lower brain weight than the male cases (t = 2.857, df = 13, p = 0.013). Doing so negatively impacted the significance of gender's effect on PC density (p = 0.068, Table 2, Test 2). However, one control male case was missing brain weight data, and by removing that case from Test 1, we were able to determine that the main difference in the significance between Test 1 and 2 was due to a loss of statistical power rather than an effect of brain weight on PC density. (Indeed, excluding this case from Test 1 resulted in a similar effect of gender, p = 0.061). Therefore, it appears that volumetric differences minimally contributed to the effect of gender on overall PC density in this investigation.

#### Lobule X is more affected in males with autism than females

From our linear mixed models descried above (Table 2, Tests 3 & 4), it was apparent that not all regions had similar PC densities. We further confirmed this phenomenon with an ANOVA (p = 1×10^−4^) using Tukey's HSD *post hoc* analysis and found that lobule X differed from the other regions in terms of PC density, most substantially from crus I & II (p<0.001) and to a lesser extent from hemispheric lobules IV–VI (p = 0.036).

As demonstrated in Figure 3, we observed that males in the autism group had a lower PC density in lobule X while females' lobule X PC densities appeared to be unaffected by autism. We hypothesized this to be a crucial component of the three-way interaction between diagnosis, gender, and region identified in the linear mixed models (Table 2, Tests 3 & 4). We further analyzed this phenomenon by performing t-tests comparing the lobule X PC density in males and females in our autism and control groups. This approach demonstrated that males in the autism group had a 31.5% lower PC density than females in the autism group (t = 3.154, df = 6, p = 0.02), while there was no difference based on gender in the control group (p = 0.768). Additionally, males in the autism group had a trend towards a 26% lower PC density than control males (t = 2.312, df = 8, p = 0.05), while there was no difference between the females in the autism and control groups (p = 0.921). We must emphasize that this is a highly preliminary finding because of the low sample size, particularly with respect to female cases. Indeed, applying a univariate generalized linear model to test for the interaction between gender and diagnosis in affecting lobule X PC density did not prove significant (p = 0.193). The observed power for this analysis was 0.24 and the effect size f was 0.36. This is a medium-sized gender by diagnosis interaction effect that would require a larger sample pool to reach significance. Further research is required to determine if this finding is reproducible in a larger study, as it may represent a unique gender-based difference in the neuropathology of autism.

We further assessed the contribution of age to our preliminary finding of a gender-based disparity in lobule X PC density in the autism group, because the females in our study were younger than the males (t = 2.35, df = 14, p = 0.036). Using generalized linear modeling with age as a covariate, we found that age only marginally affected the significance of this finding (F = 6.881, p = 0.047).

#### Volumetric differences may account for the observed gender difference in lobule X Purkinje cell density

The disparity between male and female brain weights in our autism group was nearly twice the expected difference (327 grams compared to 186 grams for the autism cases cataloged in the ATP database) [http://www.atpportal.org], which may be a reflection of the small sample size available for this study. Furthermore, PC density in lobule X was correlated with brain weight in the autism group (R^2^ = −0.67, p = 0.014). Brain weight is a correlate of brain volume [39], and thus we performed a generalized linear model with brain weight as a covariate to assess its contribution to the observed gender difference in lobule X PC density. Indeed, eliminating the effect of brain weight impacted the significance of the difference between male and female lobule X PC density in the autism group (p = 0.642), as well as the difference between males with autism compared to control males (p = 0.117).

Additionally, MRI data was available for the autism cases in our study and we used these to obtain cerebellar volume measurements. Similar to what we observed with brain weight, lobule X PC density in the autism group strongly correlated with cerebellar volume (R^2^ = −0.80±0.02, p = 0.008). After correcting the generalized linear model for covariance with cerebellar volume, we saw a similar loss of significance in the difference between male and female lobule X PC density in the autism group (p = 0.730).

In light of these findings, a gender-based disparity in lobule X volume likely contributes to the PC density differences observed in this region. This is in contrast to the findings of reduced PC density in crus I & II in autism, for which there was no observed difference in the significance following a correction for brain weight (p = 0.025 and p = 0.017). We also noticed in our linear mixed model that including brain weight as a covariate marginally increased the significance of the three-way interaction between gender, diagnosis, and region (p = 0.005, Table 2, Test 4). Therefore, this volumetric contribution to the PC density differences observed in the autism group is limited to lobule X.

### Behavioral Correlation with Purkinje Cell Density

We examined our PC density estimates looking for possible correlations with behavioral measures obtained from the ADI-R. The ADI-R is comprised of 93 questions scored on an ordinal scale, which are combined into domain scores for the three core autism symptom domains (communication, social interaction, and restricted/repetitive behavior). However, these domain scores compound the intrinsic inter-rater variance of each ADI-R question, thus diminishing the power to determine relationships between neuropathological findings and specific behavioral symptoms. Therefore, we performed regression analysis on a selection of 7 specific ADI-R questions that were recently reported to associate strongly with autism diagnosis [Presentation by Dennis Wall, “Shortening the Behavioral Diagnosis of Autism Through Artificial Intelligence and Mobile Health Technologies,” Autism Consortium 2011 Symposium, Boston, MA]. We limited our regression analyses to these specific behavioral variables and performed false discovery rate analysis (as detailed in [37]) to reduce the probability of a type I statistical error.

**Figure 5:**
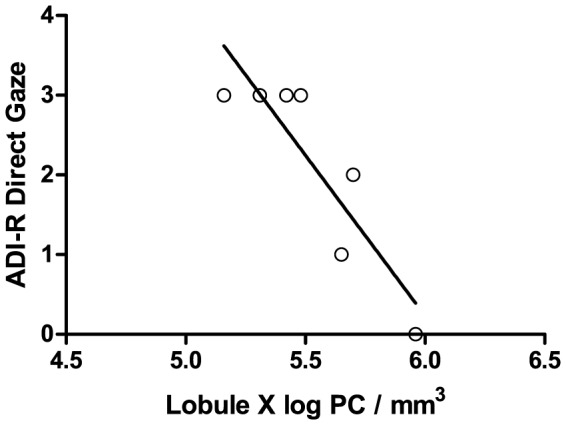
Lobule X PC Density is Associated with Direct Gaze. Lobule X PC density in the autism group negatively correlated with ADI-R question 50, which assessed the social and communicative use of eye contact (R^2^ = −0.75±0.04, p = 0.012). Higher scores on the ADI-R correspond with increased symptom severity. One case from the autism group was lacking sufficient ADI-R data to be included in this analysis.

In our initial analysis of ADI-R domain scores, we did not observe a significant association between PC density and any of the domain scores. However, in our analysis of specific questions from the ADI-R, we did identify a plausible association between lobule X PC density and social/communicative use of direct eye gaze (R^2^ = −0.75±0.04, p = 0.012) (Fig. 5). Higher scores on the ADI-R correspond with increased symptom severity. Therefore, lower lobule X PC densities were associated with greater impairments in social eye contact. Utilizing false discovery rate analysis, this association failed to meet the cutoff for significance (which was p = 0.009); however, due to known involvement of this region in regulating eye movement [20], we feel this observation merits further investigation.

As we had done above, we utilized generalized linear models with brain weight or cerebellar volume as a covariate to determine if tissue volume contributed to the observed association between lobule X PC density and direct eye gaze. Both of these covariables were found to substantially reduce the significance of the association (p = 0.072 for brain weight and p = 0.171 for cerebellar volume). Therefore, tissue volume represents a significant component of the observed association between lobule X PC density and the social and communicative use of direct gaze.

### Analyses of Clinical Covariables and Potential Confounding Factors

Potential confounding factors and clinically relevant covariables such as cerebellar hemisphere, PMI, postmortem fixation time, epilepsy, MR, developmental regression, lack of verbal development, delayed attainment of motor milestones, and complications during pregnancy were tested to determine their effects on PC density in crus I & II or lobule X, and none were found to have a significant effect. Similarly, none of these factors were found to impact the association between lobule X PC density and direct gaze.

## Discussion

### Case Demographics

Cerebellar pathology was first proposed as a potential contributor to autism symptomatology in 1968 [40], and the first qualitative report of lower PC density was published in 1980 [10]. To date, PC density assessments have been reported from 45 cerebella of individuals with autism, 30 of which demonstrated a lower PC density, most prominently reported in the posterolateral hemispheres where crus I & II are located [3]–[10], [25], [26]. Apart from the most recent studies, these reports have been criticized as being semi-quantitative at best, and the sample selection has not been representative of the demographics of the overall autism population as currently defined. For example, 87.5% of cases analyzed in prior reports (for which data is available) exhibited MR compared with the recently reported 41% in the autism population [27], and 57.9% of these cases exhibited epilepsy compared with the recent estimate of 38% in the autism population [28]. Furthermore, only 6 cases were females, despite the estimated 4.5∶1 male-to-female ratio of autism's prevalence [27]. This has prevented gender-based comparisons of autism neuropathology in the past. For this study, we included 3 female cases with autism and 3 female controls as a preliminary assessment of the effect of gender on PC density in autism. It is difficult to rule out the contribution of factors such as epilepsy or MR to lower PC density based on the data from prior neuropathological investigations of autism. The current study was thus designed with a sample population more representative of the overall autism population, with 38% of samples obtained from subjects with MR ranging from mild to severe, and 38% of samples having documented epilepsy.

### Stereological Estimation of Purkinje Cell Density

**Figure 6:**
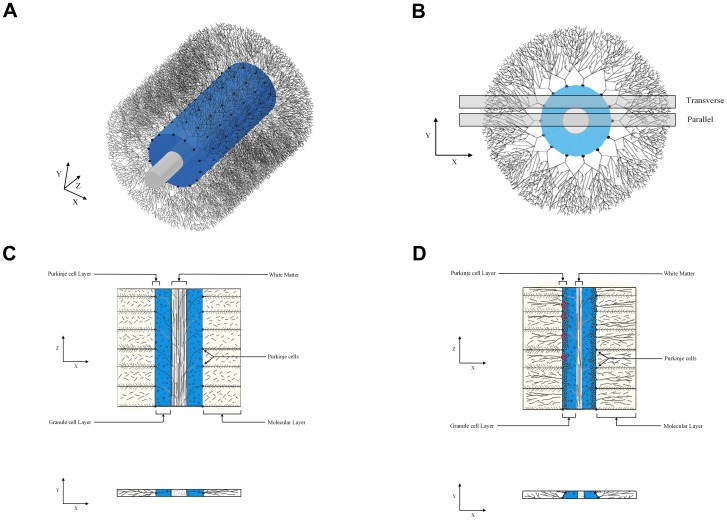
Models of PC Arrangement. These figures represent the importance of estimating PC density in 3-dimensional space rather than along a 1-dimensional line (PC/mm^3^ rather than PC/mm). 7a and 7b display two views of a 3-dimensional model of a cerebellar folium, in which PCs (black) are arranged in a monolayer apposed to the granule cell layer (blue) that surrounds a central white matter tract (white). 7b displays two slices, one perfectly parallel to a plane of PCs, and the other, more realistically, transverse to this plane. 7c and 7d are cartoons demonstrating the PC arrangement within the parallel and transverse slice, respectively. Notice in 7d the PC layer is thicker, the degree to which depends on the slice position. Also notice that the selection of which PCs lie perfectly along a line is ambiguous (a few examples have been circled in red). This ambiguity introduces human error when performing an estimate of PC/mm that is eliminated in 3-dimensional estimates of PC/mm^3^ as are utilized in this study.

**Figure 7:**
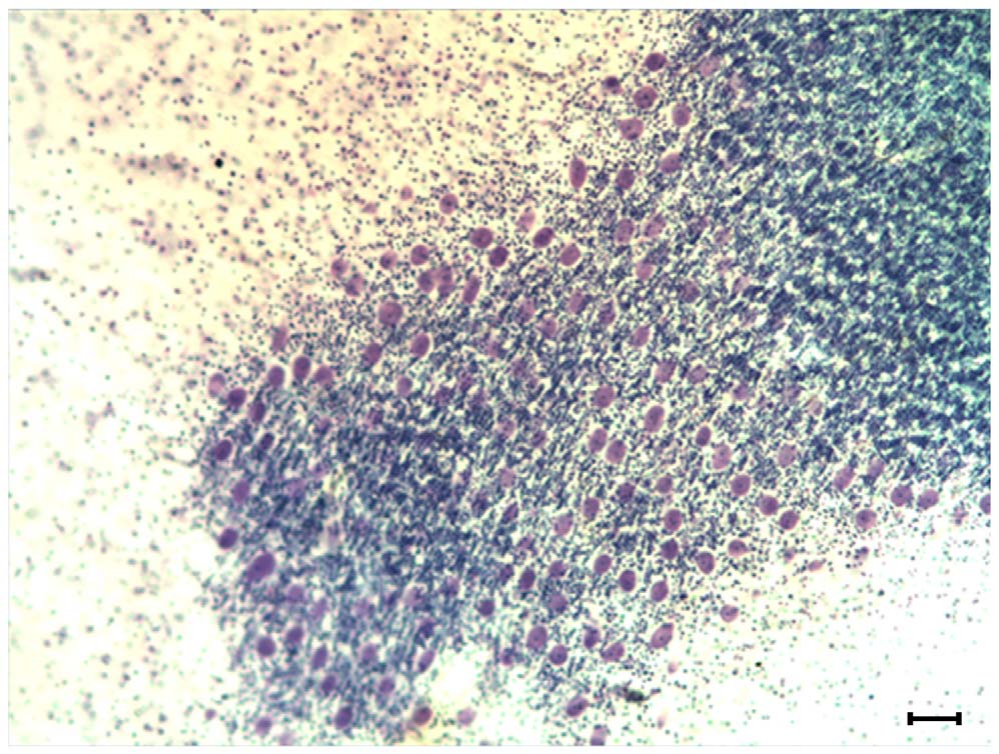
Photomicrograph of Transverse Section. This photomicrograph is an illustrative example of the arrangement of PCs on a transverse section. The PCs (purple) go out of focus to the left as the PC layer curves through the depth of the tissue section. The granule cell layer (blue) is visible below the PC layer, the molecular layer surrounds the PC layer (seen here as sparsely stained space), and the white matter tract is out of view to the right. Nissl-stained section from the celloidin collection used in this study, reference bar = 100 µm.

The determination of PC density in the cerebellum is challenging, primarily due to complications in defining the anatomical boundaries of the PC layer and selecting a reference volume. A traditional approach has been to quantify PC density by drawing a line through the PC layer and counting the number of PC per unit length. Past studies using this approach have shown markedly variable density estimates in healthy controls from as little as 1.6 PC/mm to as many as 11 PC/mm [41]–[49]. An inherent problem in placement of this line within the PC layer is the large number of subjective determinations. Factors contributing to this subjectivity include: the variable horizontal position of the PCs within this layer, the markedly convoluted folding of the layer through which the stereologist must draw a curved line, the difficulty in determining the layer's anatomical boundaries at sites where the PCs are sparse or absent, and the changing width of the layer as viewed on obliquely cut sections (Fig. 6, 7). These considerations, along with the very narrow width of the human PC layer (1 to 2% of the width of a cerebellar folium), create problems for the accurate measurement of the PC layer volume and thus for its use as a reference volume to measure the density of PCs.

In order to obviate these issues and provide a more reliable estimate of PC density, we sampled from the entirety of each region of interest, within clear anatomical boundaries, and thus included the subcortical white matter of each region. With this strategy we were able to eliminate over 50% of the variability in our PC density estimates (compare to Whitney et el. [9]). Further, our measurements of PC/mm^3^ closely agree with recently published estimates obtained using a novel stereological probe designed to better assess the density of objects like PCs that are distributed within a limited and convoluted portion of the region of interest [50].

Neuronal density is an important morphometric measure in neuropathology, despite the inherent variability that occurs as a consequence of differential tissue shrinkage in each case. In this study, tissue shrinkage resulting from tissue processing varied only marginally from 44% to 52% according to the pathologists' reports and did not differ between groups. An alternative stereological strategy is to quantify total neuronal number using the Optical Fractionator probe, but this requires 100% of the assayed region to be pristinely represented in the sampled slides [34]. Neuronal density measures are distinct from estimates of total number however, in that they reflect the spacing of neuronal somas and neuropil in a tissue, a property that has a number of physiological implications [51], [52].

### Implications of Cerebellar Neuropathology in Autism

The results of the present investigation corroborate prior reports of lower PC density in autism, and using a more precise and thorough method of quantification, demonstrate that lower PC density is most prominent in crus I and II, which constitute lobule VIIa of the posterior lobe. Lobule VIIa is intimately involved with numerous neocortical areas and has been directly implicated in non-motor functions in humans. In a report of 156 patients with cerebellar damage, 100% of patients with crus I lesions presented with attention impairments, and 100% of patients with crus II lesions presented with impairments in visuospatial memory and verbal memory (with some overlap between the two regions' functions) [53]. Further, a recent fMRI study demonstrated involvement of lobule VII in auditory working memory in nonhuman primates [54]. Anatomical and comparative anatomical studies have demonstrated a strong relationship between lobule VII and the cerebral cortex. Comparative studies highlight a relationship between the volume of the prefrontal cortex and that of lobule VII, with a pronounced elaboration of VIIa in human evolution in concert with frontal cortical expansion [55]. Lobule VIIa is reciprocally connected with the prefrontal cortex, an executive function/working memory area, as well as the posterior parietal cortex, a multimodal processing area [12], [56], [57]. Recent studies employing resting state fMRI have further demonstrated functional connectivity between lobule VIIa and these cortical areas [58], [59]. These prefrontal and posterior parietal cortices comprise a Frontoparietal Attention Network (FAN), which is postulated to play an important role in processing the salience of environmental cues [60]. The prefrontal cortex is involved in a variety of working memory and executive functions that have shown impairment in individuals with autism [61]–[65]. Further, it is important to note that in normal brain development, lobule VII displays unique features that distinguish it from other cerebellar lobules [66]–[69]. An example of the distinct developmental trajectory of lobule VII neurons was demonstrated in a mouse model of immune activation at mid-gestation, in which a specific reduction in PC density in lobule VII was observed but neighboring regions were unaffected [70].

We have also identified a potential sexually dimorphic effect within lobule X. Lobule X has been associated with vestibular regulation as well as coordination of gaze [13]–[18]. The lobule X PC density was found to be lower in males with autism as a result of increased tissue volume (Fig. 3). Furthermore, this effect appeared to correlate with the ability to properly employ social and communicative use of eye gaze, as assessed by the ADI-R (Fig. 5). This correlation between lobule X PC density and social eye gaze failed to reach the cutoff for significance imposed by false discovery rate analysis, but in light of the role of lobule X in modulating eye movement, the association is plausible [20]. These preliminary findings will require further investigation as this is the first report of the association between lobule X PC density and the social employment of eye contact. Further, we recommend that future investigations analyze the flocculus and nodulus within lobule X separately, as these regions have functional distinctions [71] and may display different pathologies.

The mechanism of the alterations in PC density noted in this study remains unexplained. As the cerebellum develops in close coordination with many other networks within the central nervous system [72]–[76], it is possible that altered PC density represents a compensatory mechanism or downstream effect of an earlier developmental pathology. In the present study, the degree to which PC density diminished with age did not differ between the autism and control groups over the age span studied (from 4 to 56 years). This lack of a notable age-related decline in PC density in the autism cases argues against a lifelong progressive loss of PCs in this disorder. The initial neuropathological studies from members of our group predicted that the decreased density of PCs observed in the cerebella of autistic individuals may be due to the loss of these neurons during the late prenatal period [5]. This prediction was based on the observation that the inferior olivary neurons in the brain stem of individuals with autism are preserved despite reduced PC densities. The intimate relationship between inferior olivary neurons and PCs is established in late gestation, and the loss of PCs in term and older aged infants is typically associated with inferior olivary degeneration. In a follow-up investigation, Whitney et al. noted in the autistic brain that there was a preservation of basket and stellate cells in areas with decreased PCs, which indicates that the PCs in these regions established their usual relationship with these interneurons in late gestation and were subsequently lost [77].

### Comments, Limitations, and Future Directions

Neuronal density can be affected by both changes in tissue volume as well as neuronal number. Therefore, we have demonstrated that the contribution of tissue volume to a subset of our findings is indeed significant.

Our study was limited by a small sample size, but each case was systematically analyzed using precise methods that allowed for the identification of consistent and potentially behaviorally relevant alterations in regional PC density in autism. Fewer female cases than would be ideal in making gender comparisons were available and thus these comparisons should be viewed as strictly preliminary. Additionally, the age range of the study was not as wide as in prior studies that identified an age-related decline in PC density. Therefore, future analyses in older patients may demonstrate a different trajectory of age-related PC decline in the autism population. Furthermore, due to the age range of our study, it was not possible to assess the early developmental processes that are believed to be crucial in understanding autism development but that occur prior to the age at which autism can be diagnosed. For a better understanding of early development, we may need to rely on animal models, as well as further advancements in *in utero* longitudinal bioimaging techniques and efforts to identify clinically relevant prenatal biomarkers [78], [79]. To better understand the relationship between neuropathology and behavioral symptomatology in autism, it will be necessary to obtain more detailed qualitative as well as quantitative behavioral measures from tissue donors.

Current investigations are underway to determine biochemical differences in crus I and II that distinguish these regions from other cerebellar regions in an attempt to better characterize the pathophysiology of autism.
